# Qualitative Analysis of Nitrogen and Sulfur Compounds in Vacuum Gas Oils via Matrix-Assisted Laser Desorption Ionization Time of Flight Mass Spectrometry

**DOI:** 10.3390/molecules29112508

**Published:** 2024-05-26

**Authors:** Morio Ueda, Jongbeom Lee, Hyeonseok Yi, Gang-Ho Lee, Yu-Jin Kim, Geon-Hee Kim, Kyeongseok Oh, Seong-Ho Yoon, Koji Nakabayashi, Joo-Il Park

**Affiliations:** 1 Kyushu Environmental Evaluation Association, Fukuoka 813-0004, Japan; 2 Department of Chemical & Biological Engineering, Hanbat National University, Daejeon 34158, Republic of Korea; 3 Carbon Materials Research Group, Research Institute of Industrial Science & Technology (RIST), Pohang 37673, Republic of Korea; h.yi@rist.re.kr (H.Y.);; 4 Department of Defense Space Engineering, Hanbat National University, Daejeon 34158, Republic of Korea; 5 Department of Chemical and Biological Engineering, Inha Technical College, Incheon 22212, Republic of Korea; kyeongseok.oh@inhatc.ac.kr; 6 Interdisciplinary Graduate School of Engineering Sciences, Kyushu University, Fukuoka 816-8580, Japan

**Keywords:** atmospheric residue, heavy oil, atmospheric residue desulfurization, molecular characterization, FT-ICR MS

## Abstract

Analysis of the heavy fractions in crude oil has been important in petroleum industries. It is well known that heavy fractions such as vacuum gas oils (VGOs) include heteroatoms, of which sulfur and nitrogen are often characterized in many cases. We conducted research regarding the molecular species analysis of VGOs. Further refine processes using VGOs are becoming important when considering carbon recycling. In this work, we attempted to classify compounds within VGOs provided by Kuwait Institute for Scientific Research. Two VGOs were priorly distillated from Kuwait Export crude and Lower Fars crude. Quantitative analysis was performed mainly using matrix-assisted laser desorption ionization time of flight mass spectrometry (MALDI-TOFMS). MALDI-TOF-MS has been developed for analyzing high-molecular-weight compounds such as polymer and biopolymers. As matrix selection is one of the most important aspects in MALDI-TOFMS, the careful selection of a matrix was firstly evaluated, followed by analysis using a Kendrick plot with nominal mass series (z*). The objective was to evaluate if this work could provide an effective classification of VGOs compounds. The Kendrick plot is a well-known method for processing mass data. The difference in the Kendrick mass defect (KMD) between C_n_H_2n−14_S and C_n_H_2n−20_O is only 0.0005 mass units, which makes it difficult in general to distinguish these compounds. However, since the z* value showed effective differences during the classification of these compounds, qualitative analysis could be possible. The analysis using nominal mass series showed the potential to be used as an effective method in analyzing heavy fractions.

## 1. Introduction

The demand for light distillates is increasing rapidly due to the growth of the population worldwide. Vacuum gas oil (VGO) is an intermediate fraction from crude oil and serves as an important source of catalytic cracking and hydrocracking [1,2,3]. VGO, known as a heavy distillate, shows difficulties during the cracking process because of the existence of its intrinsic heteroatoms, such as sulfur and nitrogen. High capital costs are needed when a new unit process is considered for treating VGO. Nevertheless, global interest in the production of ultra-low-sulfur fuels has grown significantly. Sulfur compounds in liquid fuels generate sulfur oxides and other pollutants during combustion, which later cause acid rain and other types of environmental pollution. Since it is important to design the facilities for removing sulfur-containing compounds in liquid hydrocarbon fuels, the characterization of heavy oil can be essential for the conversion of heavy fractions into low-boiling-point fractions. Until recently, low-boiling-point fractions such as atmospheric gas oil have been analyzed in detail using capillary gas chromatography (GC) [4,5,6,7,8,9]. However, owing to the low volatility of high-molecular-weight compounds, it is difficult to analyze heavier distillates via GC. Fourier transform ion cyclotron resonance mass spectrometry (FTICR-MS) is often used for analyzing heavier materials and can clarify the structure of high-boiling-point fractions [10,11,12,13,14,15,16,17,18,19,20,21,22,23,24,25,26,27]. FTICR-MS is a powerful method for analyzing heavy oils, such as atmospheric residue, vacuum residue and asphaltene. Meanwhile, conventional analyses for VGO compounds using 1D and/or 2D GC mass spectrometry (GC-MS) [28,29,30] have been reported. Chen et al. [12] reported that six basic nitrogen compounds, N_1_ (a molecule containing one nitrogen atom), N_1_O_1_, N_1_O_1_S_1_, N_1_O_2_, N_1_S_1_ and N_2_, were identified using positive-ion mass spectra, while non-basic nitrogen compounds, N_1_, N_1_O_1_, N_1_S_1_ and N_2_, are characterized by negative-ion mass spectra. Among these nitrogen compounds, N_1_-class species are predominant. FTICR-MS data indicated that the basic N_1_ class species are pyridines, quinolines and benzoquinolines, and most non-basic N_1_ class species are derivatives of benzocarbazoles. Schaub et al. [11] reported that favorable core structures were identified as monosulfur compounds (e.g., thiophenes, benzothiophenes and dibenzothiophenes). The most abundant S_1_ class species seems to be benzothiophenes and dibenzothiophenes. Even though FTICR-MS showed effectiveness in a detailed speciation of a heavy fraction, the equipment’s installation and operation costs were highly expensive.

In this work, we attempted to classify compounds in VGOs via matrix-assisted laser desorption ionization time of flight mass spectrometry (MALDI-TOF-MS). The MALDI-TOF method disperses the target sample uniformly in a matrix that absorbs the wavelength of an ultraviolet laser (nitrogen laser light: 337 nm) and irradiates the laser. When the laser irradiates the matrix, the laser light is converted into thermal energy. At this time, the matrix is rapidly heated and vaporized, and at the same time, the sample surrounded by the matrix also receives energy from the matrix and vaporizes. This ionizes, accelerates and detects the sample in the TOF/MS electric field space. The TOF/MS analyzer offers high sensitivity and selectivity for wide-range screening. It provides accurate measurement of ionizable component mass with few mass errors. This technique is ideal for both non-target and post-target analysis. Since the sample is ionized via the matrix, it is possible to measure a sample (protein, etc.) that is sensitive to heat and easily decomposed without causing fragmentation. This method can measure up to the highest-mass region compared with the mass spectrometry methods currently used, and can measure the molecular weight distribution and perform structural analysis at the same time and in a short time [31]. Although many scientists have identified compounds using both MALDI-TOF-MS and FTICR-MS [32,33], this work explores the sole employment of MALDI-TOF-MS, mainly to identify sulfur compounds and nitrogen compounds. It may bring up a fundamental question: is it possible to classify and identify compounds using MALDI-TOF-MS only? In general, it is difficult to analyze low-molecular-weight compounds with MALDI-TOF-MS, unlike the case for analyzing high-molecular-weight compounds. As reported, matrix selection is one of the most important aspects of MALDI-TOF-MS. This work proposes the classification of compounds in VGO feeds as well as hydrodesulfurization-treated VGOs by selecting the appropriate matrix. The analytical procedure was performed after pretreating samples. The Kendrick mass plot was employed to analyze sulfur and nitrogen in detail.

## 2. Experimental

### 2.1. VGO Feeds and Their HDS Reaction

Two different VGOs were used as received from the Kuwait Institute for Scientific Research. Two VGO samples were obtained from Kuwait Export crude and Lower Fars crude, named KEC-VGO and LFC-VGO, respectively. The boiling points of KEC-VGO and LFC-VGO were 350–538 °C and 350–523 °C, respectively. In case of HDS products, two VGOs were treated in a 150 mL batch autoclave (RX Engineering Co. Ltd., Anyang-si, Republic of Korea), equipped with a magnetic stirrer rotating at 300 rpm in the laboratory. Each 30 g VGO feed was treated at 370 °C for 0.5 h under an initial H_2_ pressure of 9 Mpa in the presence of 3 g of CoMo/Al_2_O_3_ catalyst. The catalyst was supplied by ART Corporation (Kawasaki, Japan).

### 2.2. Solvent Fractionation

**Figure 1:**
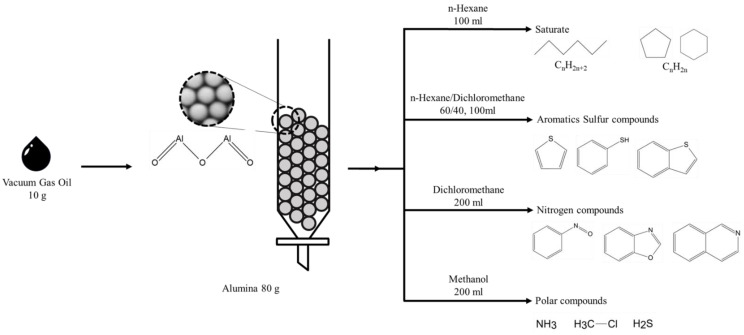
Separation method for VGO.

Sulfur and nitrogen compounds in both VGOs and their HDS products were fractionated according to an established procedure [1]. The fractionation procedure is shown in Figure 1 [34]. Activated neutral alumina (80 g) was dried at 180 °C for 4 h under vacuum conditions and then packed into a glass column (30 mm i.d. and 600 mm long). Each VGO (approximately 10 g) was placed on the top of the column. The extraction sample was eluted first with 100 mL of n-hexane for the saturated hydrocarbon fraction, and with 100 mL of n-hexane/dichloromethane (60/40 *v*/*v*) for the aromatic and sulfur compound fractions. The nitrogen compound and polar fraction were eluted by 200 mL of dichloromethane and 200 mL of methanol, respectively. The solvent of each fraction was evaporated under vacuum conditions, and the saturated hydrocarbon fraction and aromatic and sulfur compound fractions were collected using a Pasteur pipet. The nitrogen fraction and polar fraction were dissolved by tetrahydrofuran (THF) and collected.

In the MALDI-TOF-MS analysis, a number of peaks were noted after sample separation, whereas there were no clear peaks in the non-separated sample. When many compounds are present in a sample, it is possible that one of them will prevent the others from being ionized, while another compound could facilitate the ionization of the others, according to a matrix-like effect. Since a crude oil consists of many compounds, it is not easy task to determine each compound’s individual effect on ionization. However, based on the appearance of peaks after separation, it is hypothesized that some compounds prevent the ionization of other compounds, as alluded to previously. In this work, separating the feed into fractions was a useful method.

### 2.3. Sample Preparation for MALDI-TOF-MS

**Figure 2:**
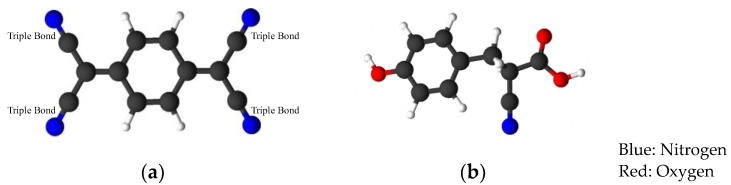
Matrix chemical structures of (**a**) 7,7,8,8,-tetracyanoquinodimethane (TCNQ) and (**b**) α-cyano-4-hydroxycinnamic acid.

**Figure 3:**
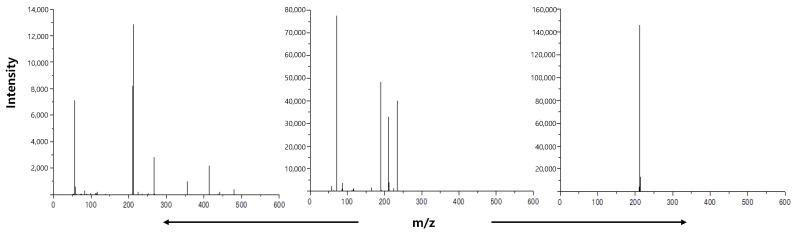
Peaks of 4,6-dimethyldibenzothiophene (**left**: without matrix; **center**: with CHCA; **right**: TCNQ).

**Figure 4:**
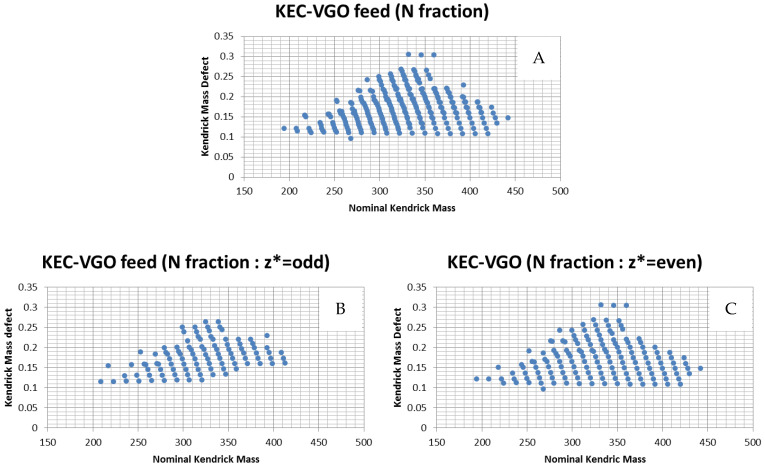
Kendrick plot of KEC-VGO feed before and after pre-sorting (**A**: without pre-sorting; **B**: z* = odd; **C**: z* = even).

In the previous study, TCNQ (2 7,7,8,8-tetracyanoquinodimethane) was introduced as the optimal matrix for the analysis of heavy fractions in MALDI-TOF-MS [35]. In this work, to verify the effectiveness of TCNQ, 4,6-dimethyldibenzothiophene was evaluated using TCNQ and α-cyano-4-hydroxycinnamic acid (CHCA). The chemical structures of both matrix chemicals are presented in Figure 2. TCNQ performs as an electron acceptor because it has four cyano groups, and tends to promote radical compounds. On the other hand, CHCA has one cyano group, one hydroxyl group and one carboxyl group, such that CHCA renders compounds not only radical, but also protonated. Thus, the ionized conditions of peaks obtained by this matrix are difficult to interpret sufficiently (Figure 3). Figure 4 shows the detected peaks. An amount of 10 g of each fraction of VGO feeds and their HDS products were dissolved in 1 mL of THF, and 10 mg of TCNQ was also dissolved in 1 mL of THF. Oil fractions were also mixed with some standard samples. The samples and matrix in THF were mixed in a 1:1 (*v*/*v*) ratio, and then 1 μL of the mixture was spotted on the sample plate. The fraction and nitrogen fraction amounts were quite small, so these fractions were dissolved in THF and collected after separation; then, the THF solutions were also mixed with standard samples and a matrix solvent in a 1:1 ratio. To obtain high-resolution data, external and internal standards (Table 1) were used. Carbazole, anthracene, 4,6-dimethyldibenzothiophene (4,6-DMDBT), pyrene, benzonaphthothiophene (BNT), perylene, coronene, dibenzochrysene (DBC) and dibenzopentacene (DBP) polyethyleneglycol (PEG) were chosen as external standards. The mixing ratio of these compounds was 40:15:100:15:10:1:5:4:5:1.5 (wt.%) [35] In addition, anthracene 4,6-DMDBT, perylene, coronene, benzocarbazole, DBC and PEG were used as internal standards. The chemical structures of the external and internal standards are presented in Table 2. Each internal standard sample amount of 1 or 10 mg was dissolved in THF. For example, a 10 μL polar fraction solution of KEC-VGO was mixed with 1 μL of coronene solution (1 mg/mL), and this mixture was in turn mixed with TCNQ solution in a 1:1 ratio and spotted on the sample plate. The mixing ratios of each fraction used in this study are shown in Table 2. Samples were analyzed using MALDI-TOF-MS (JMS-S3000; Japan Electron Optics Laboratory, Tokyo, Japan). In the polar fraction of KEC-VGO, high-resolution data were obtained from external and internal standard samples. For example, the theoretical mass values of benzocarbazoles, benzoacridines, dibenzocarbazoles and tetrahydrodibenzoacridines are 273.15120, 285.15120, 295.13555 and 297.15120, and the mass values measured using the external and internal standard methods are 273.15128, 285.15112, 295.13563 and 297.15122, respectively.

### 2.4. Kendrick Plot and Nominal Mass Series (z*)

It is known that numerous isomers make it difficult to identify each molecular species in the heavy fraction. The Kendrick plot was proposed to analyze heavy fractions, and formed the basis of the Kendrick mass defect (KMD) plot, where the KMD can be plotted against the nominal Kendrick mass (NKM) [36]. The advantage of the Kendrick plot is that members of a homologous series have identical KMD values, and the KMD has a positive correlation with the double-bond equivalent (DBE). The KMD plots are generated by multiplying the m/z of MALDI-TOF-MS data by 14.00000 and dividing the result by 14.01565, while the NKM is obtained by rounding the Kendrick mass value to the nearest integer:

Kendrick mass = *m*/*z* × 14.00000/14.01565

Nominal Kendrick mass (NKM) = the nearest integer to the Kendrick mass value

The KMD can be obtained as follows:

Kendrick mass defect = NKM − Kendrick mass

To process high-resolution mass spectral data, a multiple sorting technique based on nominal mass series (z*) is effective, as is KMD [5]. The difference in KMD between C_n_H_2n−14_S and C_n_H_2n−20_O is only 0.0005 mass units. Therefore, it is not easy to distinguish between oxygen compounds and sulfur compounds, but the z* for C_n_H_2n−14_S and C_n_H_2n−20_O is −10 and −4, respectively, where z* is defined as follows:

z* = the modulus of (nominal mass/14) − 14

The z* value is between −14 and −1 and is related to the valence number of the heteroatom. For example, the valence number of a sulfur atom is 2, so the z* of S-containing compounds is even. On the other hand, the z* of N-containing compounds is odd because the valence number of a nitrogen atom is 3. However, in the case of a compound with one sulfur atom and one nitrogen atom (S_1_N_1_ class species), the z* is odd because the total valence number is odd. In other words, compounds with odd z* values are N_1_, N_1_S_1_, N_1_O_1_, N_1_S_1_O_1_, N_1_S_2_, N_1_ O_2_, N_3_ and so on, while S_1_, O_1_, S_2_, O_2_, S_1_O_1_, N_2_, N_2_S_1_ and N_2_O_1_ have even z* values. The z* values are well separated to distinguish between compounds with similar KMDs. Kendrick plots can be relatively complicated due to the presence of many compounds in oil, but clarity can be achieved by pre-sorting (Figure 3). Thus, masses were sorted according to the z* value before KMD sorting. In this study, z* = odd for polar and nitrogen fractions, and even for aromatic and sulfur fractions.

### 2.5. GPC-UV Measurement

GPC-UV analysis was performed using a 1200 infinity series GPC instrument (Agilent technologies, Santa Clara, CA, USA) equipped with Diode Array Detector (DAD at 261 nm), an injection valve with a 300 μL loop. An LF-404 column (4.6 mm × 250 mm, Shodex, Tokyo, Japan) with 6 μm particle size ‘multi-pore’ gel as the stationary phase was used for high-resolution spectroscopy, and the LF-G column (4.6 mm × 10 mm, Shodex, Japan) was used as a guard column. Polystyrene commercial standard samples (SL-105, Shodex, Japan) were used for calibration. The mobile phase was THF in order to enhance the solubility of oil samples. Each standard sample was prepared by diluting a nominal concentration of 0.1% in THF and filtered by using a membrane filter (0.45 μm, Teflon, Millipore, St. Louis, MO, USA) in this study. The oil samples were also dissolved and filtered. The column temperature was stabilized at 40 °C. The THF solvent flowed overnight for stabilization. The flow rate was fixed at 0.2 mL min^−1^, and a 5 μL aliquot of the oil sample was analyzed.

## 3. Results and Discussions

### 3.1. Classification of Compounds in KEC-VGO Feed and HDS Products

#### 3.1.1. Polar and Nitrogen Fraction in KEC-VGO Feed and HDS Products

**Figure 5:**
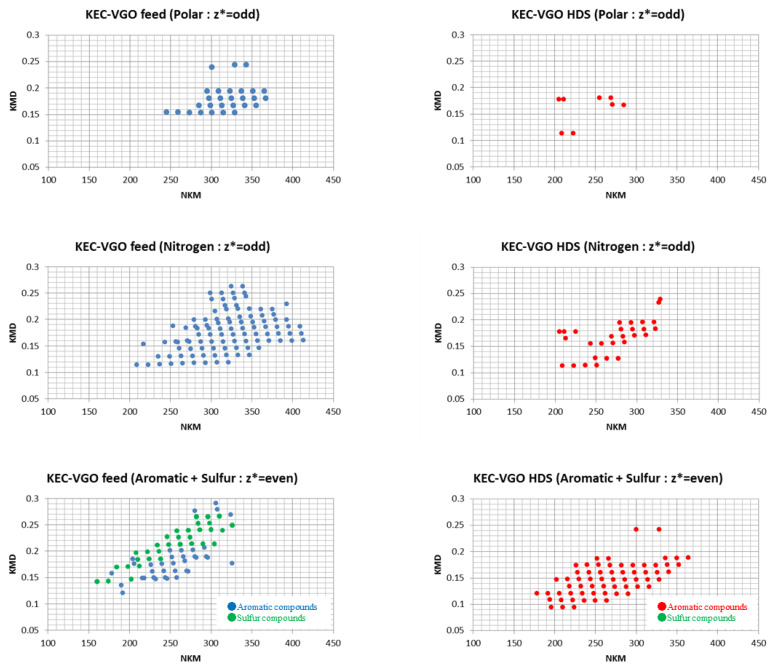
Kendrick plot of each fraction in KEC-VGO feed and HDS product.

As mentioned in the experimental section, pre-z* sorting can be an effective method to classify the type of compounds present. The m/z of each fraction in KEC-VGO was determined based on the z* number and plotted on a Kendrick plot (Figure 5). In the case of odd z* values, many N_1_ species were classified. In particular, in the polar and nitrogen fraction, N_1_ class species were dominant and few S_1_N_1_ class species were present (Table 3). N_1_ class species are expected to have a carbazole or acridine structure. Based on the KMD plot, C_n_H_2n−15_N, C_n_H_2n−17_N, C_n_H_2n−19_N, C_n_H_2n−21_N, C_n_H_2n−23_N, C_n_H_2n−25_N and C_n_H_2n−27_N are carbazole, acridine, tetrahydrobenzoacridine, benzocarbazole, benzoacridine, tetrahydrodibenzoacridine and dibenzocarbazole, respectively. N_1_ class species were confirmed in the z* = odd polar and nitrogen fraction group, and the states of these compounds may be radical. Carbazoles and acridines have some benzene rings, so even if an electron in the pie orbital is removed, carbazoles and acridines in a radical state should be stable due to the delocalization of pie electrons. The function of the TCNQ matrix is to achieve a radical state, but not all of the compounds change into a radical state; some are protonated or become anions. N-containing compounds could be protonated. Because the nitrogen atom is a Lewis base, a proton can interact with the nitrogen atom in N-containing compounds. Thus, in the case of compounds with an N atom, not only is a radical structure detected, but so too is a protonated structure. If a proton is added to N-containing species, the z* value changes. For example, the z* value of radical benzoacridines is −9, but that of protonated benzoacridines is −8. Thus, in the polar and nitrogen fractions, nitrogen species could be detected in the even z* group, but it is possible to separate protonated compounds according to the pre-z* sorting step.

After the HDS reaction, the values of KMD decreased in both fractions, with compounds of a larger molecular weight being particularly affected. This shows that compounds of a high DBE are hydrogenated into products of a low DBE, and the molecular size of long alkyl chains of compounds is reduced. In the nitrogen fraction, after the HDS reaction, acridines and carbazoles were detected, and some compounds were confirmed within the 0.15 to 0.2 KMD range, but could not be classified.

#### 3.1.2. Aromatic and Sulfur Fraction in KEC-VGO Feed and HDS Products

Several aromatic compounds and sulfur-containing compounds were confirmed in the aromatic and sulfur fractions. In the plot of aromatic and sulfur fractions, the KMD of sulfur compounds is larger than that of aromatics compounds. The formulas of the sulfur compounds are C_n_H_2n−12_S, C_n_H_2n−16_S, C_n_H_2n−18_S, C_n_H_2n−20_S, C_n_H_2n−24_S, C_n_H_2n−26_S, C_n_H_2n−28_S and C_n_H_2n−30_S, and the structures corresponding to these formulas are tetrahydrodibenzothiophene, dibenzothiophene, acenaphthenothiophene, acenaphthlemothiophene, naphthenephenanthrenothiophene, pyrenothiophene, chrysenothiophene and cholanthrenthiophene, respectively. Aromatic compounds were confirmed in a small KMD area; the formulas are C_n_H_2n−16_, C_n_H_2n−18_, C_n_H_2n−20_, C_n_H_2n−22_, C_n_H_2n−24_, C_n_H_2n−26_, C_n_H_2n−28_ and C_n_H_2n−30_, corresponding to octahydronaphthacene anthracene, tetrahydrobenzoanthracene, pyrene, chrysene, tetrahydropicene, perylene and dicyclopentapyrene, respectively. After the HDS reaction, a number of sulfur-containing compounds were reduced and peaks of aromatic compounds with small KMDs appeared. This result means that under hydrogen pressure, hydrogenation occurs alongside the HDS reaction on the CoMo catalyst. Interestingly, the molecular weight of some compounds in the HDS product was larger than that of some compounds in the feed.

### 3.2. Classification of Compounds in LFC-VGO Feed and HDS Products

#### 3.2.1. Polar and Nitrogen Fraction in LFC-VGO Feed and HDS Products

**Figure 6:**
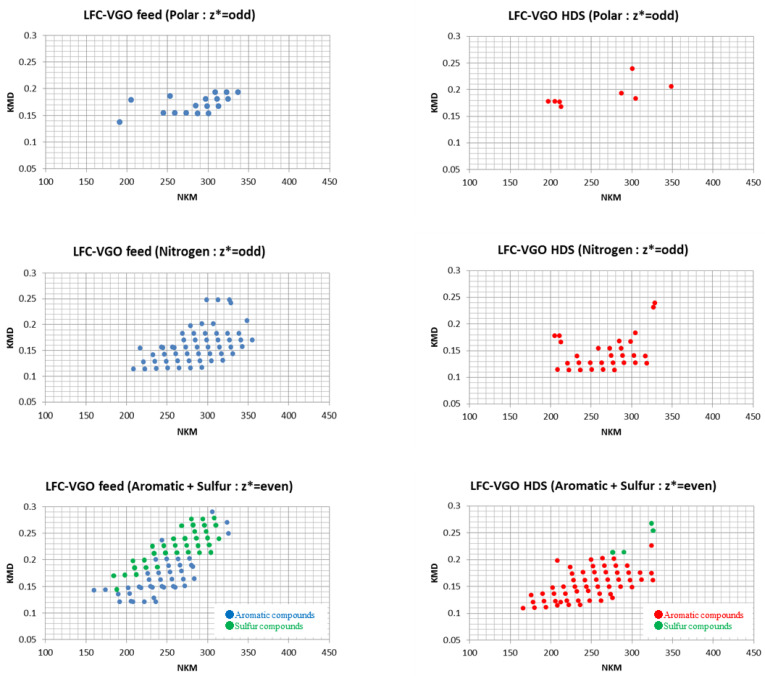
Kendrick plot of each fraction in LFC-VGO feed and HDS product.

As mentioned in the experimental section, pre-z* sorting is an effective method to classify the type of compounds present. The m/z of each fraction in LFC-VGO was determined based on the z* number and plotted on a Kendrick plot (Figure 6). As well as KEC-VGOs, some N_1_ species were confirmed in the polar fraction of LFC-VGO, and the types of compounds were similar. However, S_1_N_1_ species could not be confirmed in the polar fraction in LFC-VGOs (Table 3). There were fewer peaks for the polar fraction of LFC-VGOs compared with that of KEC-VGOs, and the carbon number of the alkyl chains was small. After the HDS reaction, benzoacridines were present, but no other compounds were classified. In the nitrogen fraction, N_1_ species were also dominant in the LFC-VGO feed, but there were fewer high-DBE species relative to the KEC-VGOs. Species with a high DBE were converted into compounds with a low DBE after the HDS reaction, and carbazoles, acridines, tetrahydrobenzoacridines, benzocarbazoles, benzoacridines and tetrahydrodibenzoacridines were detected. In particular, acridines and carbazoles in the HDS product of the LFC-VGOs had long alkyl chains compared with those of the HDS product compounds of the KEC-VGOs.

#### 3.2.2. Aromatic and Sulfur Fractions in LFC-VGO Feed and HDS Products

**Figure 7:**
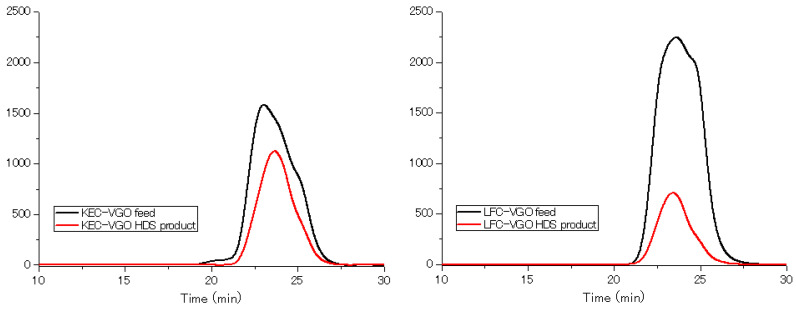
Results of GPC analysis for (**left**) KEC-VGO and (**right**) LFC-VGO. Black line: feed; red line: HDS product.

Aromatic and sulfur fractions in LFC-VGO showed similar KEC patterns. Sulfur compounds have large KMD values and aromatics have small KMD values, but some compounds were plotted in the low KMD range. In the HDS products, a few benzonaphthothiophenes, chrysenothiophenes and cholanthrenothiophenes were detected. As for the other compounds, octahydronaphthacene, anthracene, tetrahydrobenzoanthracene, pyrene, chrysene, tetrahydropicene and perylene were confirmed to be present (Table 3), similar to the HDS products of KEC-VGO. In both KEC-VGO and LFC-VGO, the number of compounds with large KMD values decreased, while compounds with small KMD values remained after the reaction. This shows that compounds with a high DBE are hydrogenated because they have some benzene rings. Benzene rings have π bonds that are easily converted into σ bonds, so the catalyst interacts with π electrons in the benzene rings and the bonds are cut under high hydrogen pressure. Compounds with abundant benzene rings have many π electrons; thus, these compounds interact with the surface of the catalyst, such that compounds with a high DBE are hydrogenated first. Interestingly, the molecular weight of some compounds in the HDS product is larger than that of certain compounds in the KEC-VGO. It is possible that compounds with a high molecular weight and high DBE cannot be ionized, or that the concentration is quite small. Gel permeation chromatography (GPC) analysis was conducted for the feed and HDS products of aromatic and sulfur fractions in the VGOs. Based on the results of the GPC analysis, the molecular size decreased (Figure 7), so the molecular weight became smaller after the HDS reaction, and some compounds with a high molecular weight and high DBE could not be ionized.

## 4. Conclusions

Prior to the application of MALDI-TOF-MS analysis, sample fractionation was performed using liquid chromatography in the presence of packed alumina as a pre-separation procedure. The spectra proved to be more effective in analyzing VGOs, as a greater number of peaks appeared after fractionation than in the non-pre-separated samples. Careful matrix selection is one of the most important aspects of MALDI-TOF/MS. In this work, 4,6-DMDBT was investigated as a standard sample to find the most suitable measurement conditions. TCNQ proved to be the best matrix as it ensured that the ionized compound was in a radical state due to its four cyano groups. In order to obtained high-resolution data, internal standard and external standard methods were evaluated and shown to be effective, as long as standard samples were used, and the mass defect was kept to a minimum. The Kendrick plot is a well-known method for interpreting mass data. The employment of nominal mass series (z*) is an effective alternative method. Since the difference in KMD between C_n_H_2n−14_S and C_n_H_2n−20_O is only 0.0005 mass units, it is not an easy task to distinguish these compounds without further refining the data. However, the z* value was different between these compounds; therefore these species can be classified on the basis of this value. Using z* values, compounds of each fraction in the VGOs were apparently classified. In the case of the polar and nitrogen fractions of both VGOs, N_1_ species were dominant and a few S_1_N_1_ species were also present. Compounds in the polar fractions of the KEC-VGO feed had longer alkyl chains compared with those of the LFC-VGO feed, and in the nitrogen fractions, alkyl chains of the KEC-VGO feed were longer than those of the LFC-VGO feed. After the HDS reaction was applied to both VGOs, compounds of a high molecular weight and high DBE were reduced in number. This supports the idea that alkyl chains were cut, and benzene rings were hydrogenated in the presence of hydrogen. Though it was not expected, there was no major difference in the plots of the aromatic and sulfur fractions between the KEC-VGO and LFC-VGO feeds. Sulfur-containing compounds corresponded to high values of KMD in the Kendrick plot, while aromatic compounds corresponded to low values of KMD. After the HDS reaction, almost all sulfur compounds were reduced and aromatic compounds appeared with a low DBE. Although the compounds were hydrogenated, the molecular weight of some species in the HDS product was larger than that of compounds dispersed in the feed. It may be possible that species of a larger molecular weight were present in the VGO, but were hardly detected due to the low concentrations or inappropriate conditions for ionization.

## Figures and Tables

**Table 1:** Chemical structures of external and internal standards.

Name	Structure	External	Internal
Carbazole	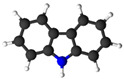 Blue: Nitrogen	O	
Anthracene	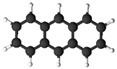	O	O
4,6-dimethyldibenzothiophene (4,6-DMDBT)	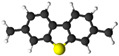 Yellow: Sulfur	O	O
Pyrene	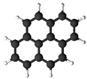	O	
benzonaphthothiophene	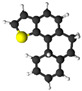 Yellow: Sulfur	O	
Perylene	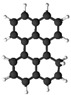	O	O
coronene	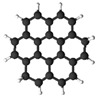	O	O
benzocarbazole	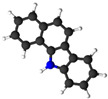 Blue: Nitrogen		O
Dibenzo[g,p]chrysene (DBC)	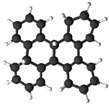	O	O
Dibenzo[a,l]pentacene (DBP)	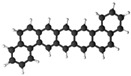	O	
Polyethylene glycol (PEG)	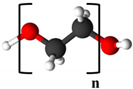 Red: Oxygen	O	O

**Table 2:** Mixing ratio of each fraction and internal standard samples.

	Fraction and Volume	Anthracene	4,6-DMDBT	Benzocarbazole	Perylene	Coronene	DBC	PEG
KEC-VGO feed	Polar 10 μL	-	-	-	-	1 μL (1 mg/mL)	-	-
	Nitrogen 10 μL	-	-	10 μL (1 mg/mL)	1 μL (1 mg/mL)	-	1 μL (1 mg/mL)	3 μL (10 mg/mL)
	Aromatics + Sulfur 10 μL (10 mg)	-	-	1 μL (1 mg/mL)	-	-	1 μL (1 mg/mL)	1 μL (10 mg/mL)
KEC-VGO HDS products	Polar 1000 μL	-	100 μL (1 mg/mL)	-	-	0.3 μL (1 mg/mL)	-	
	Nitrogen 10 μL	-	5 μL (1 mg/mL)	-	-	-	1 μL (1 mg/mL)	1 μL (10 mg/mL)
	Aromatics + Sulfur 100 μL (100 mg)	1 μL (1 mg/mL)	-	-	-	1 μL (1 mg/mL)	1 μL (10 mg/mL)	
LFC-VGO feed	Polar 10 μL	-	-	-	1 μL (1 mg/mL)	-	-	-
	Nitrogen 10 μL	-	-	10 μL (1 mg/mL)	-	-	3 μL (1 mg/mL)	3 μL (10 mg/mL)
	Aromatics + Sulfur 10 μL (10 mg/mL)	1 μL (1 mg/mL)	-	-	-	-	1 μL (1 mg/mL)	1 μL (10 mg/mL)
LFC-VGO HDS products	Polar 1000 μL	-	100 μL (1 mg/mL)	-	-	0.3 μL (1 mg/mL)	-	-
	Nitrogen 10 μL	-	10 μL (1 mg/mL)	-	-	-	1 μL (1 mg/mL)	-
	Aromatics + Sulfur 100 μL (10 mg/mL)	-	50 μL (10 mg/mL)	-	-	0.5 μL (1 mg/mL)	0.1 μL (10 mg/mL)	-

**Table 3:** Expected structure of polar, nitrogen and aromatic + sulfur fraction in the KEC-VGO feed and HDS products. DBE represents the double bond equivalent.

KEC-VGO				
Fraction	Formula (DBE)	Expected Structure	Feed	HDS Products
Polar	CnH2n-25SN (14)	-	O	
	CnH2n-27N (15)	Dibenzocarbazole	O	
	CnH2n-25N (14)	Tetrahydrodibenzoacridine	O	O
	CnH2n-23N (13)	Benzoacridine	O	O
	CnH2n-21N (12)	Benzocarbazole	O	
	CnH2n-15N (9)	Carbazole		O
Nitrogen	CnH2n-25SN (14)	-	O	
	CnH2n-23SN (13)	-	O	
	CnH2n-21SN (12)	-	O	
	CnH2n-19SN (11)	-	O	
	CnH2n-27N (15)	Dibenzocarbazole	O	
	CnH2n-25N (14)	Tetrahydrodibenzoacridine	O	
	CnH2n-23N (13)	Benzoacridine	O	
	CnH2n-21N (12)	Benzocarbazole	O	
	CnH2n-19N (11)	Tetrahydrobenzoacridine	O	
	CnH2n-17N (10)	Acridine	O	O
	CnH2n-15N (9)	Carbazole	O	O
Aromatic + Sulfur	CnH2n-30S (15)	Cholanthrenthiophene	O	
	CnH2n-28S (14)	Chrysenothiophene	O	
	CnH2n-26S (13)	Pyrenothiophene	O	
	CnH2n-24S (12)	Naphthenephenanthrenothiophene	O	
	CnH2n-30 (16)	Dicyclopentapyrene	O	
	CnH2n-20S (11)	Benzonaphthothiophene	O	
	CnH2n-28 (15)	Perylene	O	
	CnH2n-18S (10)	Acenaphthenothiophene	O	
	CnH2n-26 (14)	Tetrahydropicene	O	O
	CnH2n-16S (9)	Dibenzothiophene	O	
	CnH2n-24 (13)	Chrysene		O
	CnH2n-22 (12)	Pyrene	O	O
	CnH2n-12S (7)	Tetrahydrodibenzothiphene	O	
	CnH2n-20 (11)	Tetrahydrobenzoanthracene		O
	CnH2n-18 (10)	Anthracene	O	O
	CnH2n-16 (9)	Octahydronaphthacene		O

## Data Availability

Data are contained within the article and Supplementary Materials.

## Supplementary Materials

The following supporting information can be downloaded at: https://www.mdpi.com/article/10.3390/molecules29112508/s1.
